# Bile Acid Sequestration Reduces Plasma Glucose Levels in *db/db* Mice by Increasing Its Metabolic Clearance Rate

**DOI:** 10.1371/journal.pone.0024564

**Published:** 2011-11-07

**Authors:** Maxi Meissner, Hilde Herrema, Theo H. van Dijk, Albert Gerding, Rick Havinga, Theo Boer, Michael Müller, Dirk-Jan. Reijngoud, Albert K. Groen, Folkert Kuipers

**Affiliations:** 1 Department of Pediatrics, Center for Liver, Digestive and Metabolic Diseases, University Medical Center Groningen, University of Groningen, Groningen, The Netherlands; 2 Department of Laboratory Medicine, Center for Liver, Digestive and Metabolic Diseases, University Medical Center Groningen, University of Groningen, Groningen, The Netherlands; 3 Nutrigenomics Consortium, TI Food and Nutrition, Wageningen, The Netherlands; 4 Nutrition, Metabolism and Genomics Group, Division of Human Nutrition, Wageningen University, Wageningen, The Netherlands; Florida International University, United States of America

## Abstract

**Aims/Hypothesis:**

Bile acid sequestrants (BAS) reduce plasma glucose levels in type II diabetics and in murine models of diabetes but the mechanism herein is unknown. We hypothesized that sequestrant-induced changes in hepatic glucose metabolism would underlie reduced plasma glucose levels. Therefore, *in vivo* glucose metabolism was assessed in *db/db* mice on and off BAS using tracer methodology.

**Methods:**

Lean and diabetic *db/db* mice were treated with 2% (wt/wt in diet) Colesevelam HCl (BAS) for 2 weeks. Parameters of *in vivo* glucose metabolism were assessed by infusing [U-^13^C]-glucose, [2-^13^C]-glycerol, [1-^2^H]-galactose and paracetamol for 6 hours, followed by mass isotopologue distribution analysis, and related to metabolic parameters as well as gene expression patterns.

**Results:**

Compared to lean mice, *db/db* mice displayed an almost 3-fold lower metabolic clearance rate of glucose (p = 0.0001), a ∼300% increased glucokinase flux (p = 0.001) and a ∼200% increased total hepatic glucose production rate (p = 0.0002). BAS treatment increased glucose metabolic clearance rate by ∼37% but had no effects on glucokinase flux nor total hepatic or endogenous glucose production. Strikingly, BAS-treated *db/db* mice displayed reduced long-chain acylcarnitine content in skeletal muscle (p = 0.0317) but not in liver (p = 0.189). Unexpectedly, BAS treatment increased hepatic FGF21 mRNA expression 2-fold in lean mice (p = 0.030) and 3-fold in *db/db* mice (p = 0.002).

**Conclusions/Interpretation:**

BAS induced plasma glucose lowering in *db/db* mice by increasing metabolic clearance rate of glucose in peripheral tissues, which coincided with decreased skeletal muscle long-chain acylcarnitine content.

## Introduction

Type 2 diabetes is a major health problem worldwide [1]. The predominant features of type 2 diabetes entail increased fasting blood glucose levels, increased plasma triglycerides and LDL-cholesterol levels, as well as disturbed peripheral glucose utilization [2]–[4]. The use of bile acid sequestrants (BAS) for lowering of LDL-cholesterol levels is well established [5]–[7]. More recently, Colesevelam HCl, a BAS, has been indicated by the FDA to improve glycemic control in patients with type 2 diabetes [8]–[10]. So far, however, the actual changes in hepatic and/or peripheral glucose metabolism upon BAS supplementation are not understood.

Bile acids are synthesized from cholesterol in the liver. Upon secretion into bile, bile acids function to emulsify fats in the small intestine. Most of the secreted biliary bile acids are reabsorbed in the ileum (enterohepatic circulation). Sequestrants interfere with the enterohepatic circulation of bile acids by binding them in the intestine, thereby inhibiting their reabsorption and promoting their fecal loss. As a consequence, the liver increases bile acid synthesis and subsequently cholesterol uptake from the circulation thereby reducing LDL-cholesterol levels [11], [12].

To date, there is a lack of understanding how BAS reduce plasma glucose levels. However, a large body of research suggests that bile acids modulate hepatic glucose metabolism via signaling pathways mediated by the nuclear receptor NRH1H4 (Fxr) in diabetes [13]–[19]. Fxr is expressed in the liver, intestine, adrenal gland and kidney [20], and it acts to inhibit *de novo* bile acid synthesis when activated by bile acids in the liver [21]. Paradoxically, both agents that inhibit *de novo* bile acid synthesis, such as bile acids themselves and synthetic Fxr ligands [14], [22], as well as agents that increase *de novo* bile acid synthesis such as BAS, were shown to reduce plasma glucose levels in diabetic mice [23], [24]. Thus, regulation of bile acid-mediated changes in blood glucose levels remains elusive.

We therefore questioned whether BAS-induced changes in hepatic carbohydrate fluxes are responsible for the observed reduction in plasma glucose levels in *db/db* mice. To test this, we treated healthy lean mice and obese, diabetic *db/db* mice with Colesevelam HCl. Applying an *in vivo* infusion protocol of stable isotopes followed by mass isotopologue distribution analysis (MIDA), we first characterized specific disruptions of whole body glucose turnover and hepatic glucose metabolism in *db/db* mice. We then tested the hypothesis that BAS restores disrupted hepatic glucose fluxes thereby mediating the previously observed reduction in blood glucose levels in diabetic mice. In view of the strong interaction between glucose and fatty acid metabolism, we additionally tested the effect of BAS on levels of lipids and intermediates of fatty acid metabolism in liver and muscle.

## Materials and Methods

### Animals and diets

Ten week old male lean C57BL/6J and obese, diabetic *db/db* mice on a C57BL6/J background (B6.Cg-*m* +/+ *Lepr^db^*/J) were purchased from Charles River Laboratories (L'Arbresle, France and Brussels, Belgium, respectively). Mice were housed in a temperature controlled (21°C) room with a dark-light cycle of 12 h each. For all animal experiments the principles of laboratory animal care (NIH publication no. 85–23, revised 1985) were followed. The Ethics Committee for Animal Experiments of the University Groningen, the Netherlands, approved experimental procedures (Experiment ID: DEC5030 University of Groningen).

One week after arrival at the animal facility, 8 *db/db* (db) and 6 lean (L) mice were put on a diet containing standard laboratory chow (RMH-B; Arie Blok, Woerden, The Netherlands) supplemented with 2% (wt/wt) Colesevelam HCl (Daiichi Sankyo, Inc., Parsippany, NJ, USA) for 2 weeks. Another 8 *db/db* and 6 lean mice remained on standard laboratory chow. Body weights and food intake were recorded every other day. Mice were fitted with a permanent catheter in the right atrium via the jugular vein, as described previously [25]. Mice were allowed to recover from surgery for 6 days.

*Materials* The following isotopes were used: 2-^13^-C glycerol (99% ^13^C atom percent excess), [1-^2^H]-galactose (98% ^2^H atom percent excess) (Isotec, Miamisburg, Ohio, USA), [U-^13^C]-glucose (99% ^13^C atom percent) (Cambridge Isotope Laboratories, Andover, Mass., USA). All reagents and chemicals used were reagent pro analysis grade. Blood spots and urine were collected on Schleicher and Schuell No. 2992 filter paper (Schleicher and Schuells, ‘s Hertogenbosch, The Netherlands). Infusates were freshly prepared and sterilized at the day before the experiment.

### Animal Experiments

The infusion experiment was performed in conscious mice, as described previously [25]. Mice were fasted for 4 hours (03:00–07:00 am) and then housed in metabolic cages to allow frequent collection of urine and blood spots on filter paper. Mice were infused with a sterile solution containing [U-^13^ C]-glucose (13.9 µmol/ml), [2-^13^C]-glycerol (160 µmol/ml), [1-^2^H]-galactose (33 µmol/ml) and paracetamol (1.0 mg/ml) at a rate of 0.6 ml/h. Before and during the experiment, small blood samples were obtained via tail bleeding to allow for the determination of plasma glucose. Blood was immediately centrifuged and stored at −20°C until analysis. Blood spots were collected on filter paper before the start of the infusion and hourly afterwards until 6 h after the start of the infusion. Blood spots were air dried and stored at room temperature until analysis. Hourly urine samples were collected on filter paper, air dried and stored at room temperature until analysis. At the end of the experiment, animals were anesthetized with isoflurane and a small blood sample was collected via orbital puncture for the determination of insulin.

Mice were allowed to recover and five days after the infusion experiment. Mice were then fasted for 7 hours (03:00–10:00 h) and terminated by heart puncture under isoflurane anesthesia. A large blood sample was collected in heparin-containing tubes, immediately centrifuged and stored at −20°C until analysis. Liver was excised, weighed, snap frozen and stored at −80°C until further analysis. Gastrocnemius and plantaris muscle as well as epididymal white adipose tissue were collected, frozen in liquid N_2_ and stored at −80°C until further analysis.

Measurement and Analysis of Mass Isotopologue Distribution Analysis by *GC-MS* Analytical procedures for extraction of glucose in bloodspots and paracetamol-glucuronide from urine filter paper strips and derivatization of the extracted compounds and GC-MS measurements of derivatives were all performed according to Van Dijk *et al* [26], [27]. The measured fractional distribution was corrected for natural abundance of ^13^C by multiple linear regression as described by Lee *et al*. [28]to obtain the excess mole fraction of mass isotopologues due to incorporation and dilution of infused labeled compounds, i.e., [2-^13^C]-glycerol, [U-^13^C]-glucose and [1-^2^H]-galactose. This distribution was used in mass isotopologues distribution analysis (MIDA) algorithms of isotope incorporation and dilution according to Hellerstein *et al*. [29] as described by Van Dijk *et al* [26], [27]. Rate of glucose disposal, metabolic clearance rate, hepatic glucose production rates as well as the hepatic glucose fluxes are part of MIDA.

### Determination of metabolite concentrations

Commercially available kits were used to determine plasma levels of insulin (Mercodia, Uppsala, Sweden), triglycerides, total cholesterol, free cholesterol and NEFA (Wako Chemicals, Neuss, Germany).

Plasma HOMA-index was calculated multiplying the blood glucose levels by the plasma insulin levels at 6 h of MIDA infusion and dividing the product by 22.5 [30]. Hepatic glycogen and glucose-6-phosphate content were determined as previously described [25]. Hepatic lipids were determined in liver homogenates by commercially available kits for triglycerides and total cholesterol (Wako Chemicals, Neuss, Germany) after lipid extraction as described by Bligh and Dyer [31]. Plasma acylcarnitines were determined according to the method of Chase *et al* [32] as described by Derks *et al*. [33]. Profiles of long-chain acylcarnitines (C16∶0, C16∶1, C18∶0, C18∶1 and C18∶2) in muscle and liver homogenates (15% (w/v) in PBS) were determined according to the method of Gates [33].

### mRNA levels

Total RNA was isolated from liver using TRI-reagent (Sigma, St. Louis, MO) according to the manufacturers' protocol. cDNA was produced as described by Plösch and coworkers [34]. Real-time PCR was performed on a 7900HT FAST real-time PCR system using FAST PCR master mix and MicroAmp FAST optical 96 well reaction plates (Applied Biosystems Europe, Nieuwerkerk ad IJssel, The Netherlands). Primer and probe sequences have been published before (www.labpediatrics.nl) and deposited in the RTprimerDB. PCR results were normalized to 18S-rRNA abundance.

### Statistics

All values are represented as mean ± standard deviation. Statistical significance was assessed using the Mann-Whitney-U-test (SPSS 12.0.1 for Windows). P-values were corrected for multiple comparison errors. Statistical significance was accepted for a p<0.05.

## Results

### Bile acid sequestration reduced plasma glucose values in db/db mice

Previously, it has been observed that Colesevelam HCl treatment lowers plasma glucose concentrations in *db/db* mice compared to untreated counterparts [24]. To confirm and elaborate these findings, basal parameters related to glucose metabolism were determined in lean and *db/db* mice treated with the bile acid sequestrant (BAS) for two weeks. As expected, BAS-treatment significantly lowered blood glucose levels of diabetic mice (Table 1). No effects of BAS on body weight, liver weight or liver weight/body weight ratio were observed (Table 1). BAS treatment had no effect on 4 h fasted plasma insulin levels in *db/db* mice, while a 30% (non-significant) reduction of plasma insulin levels occurred at the end of the MIDA infusion experiment (Table 1). The HOMA-index was significantly improved in sequestrant-treated diabetic mice compared to untreated counterparts. Moreover, we observed a non-significant decrease in plasma NEFA levels, a more than 50%, however non-significant, reduction, in plasma 3-hydroxybutyrate levels with no apparent effect on plasma lactate concentrations (Table 1). Untreated lean and *db/db* mice displayed similar plasma triglyceride levels, which decreased in both models upon BAS-treatment. Plasma cholesterol levels were slightly, but significantly, elevated in untreated *db/db* mice compared to untreated lean mice: we hypothesize this to be due to decreased activity of FXR which leads to an increase in hepatic ApoA1 expression as shown in a previous study [35]. Apparently, this effect was stronger in *db/db* mice than in lean mice because no significant effect was observed in the latter. We hypothesize this to be due to decreased activity of FXR which leads to an increase in ApoA1 confirming data in a previous study. Apparently, this effect was stronger in db/db mice than WT because no significant effect was observed in WT mice. Consistent with our previous observations [24], liver triglyceride contents were significantly increased in sequestrant-treated lean and *db/db* mice compared to controls, (Table 1). Diabetic mice had significantly higher hepatic glycogen contents compared to lean mice while, surprisingly, hepatic glucose-6-phosphate contents was decreased in the *db/db* genotype, BAS treatment had no effect herein (Table 1). Additionally, hepatic levels of free fatty acids were increased ∼10-fold in *db/db* mice with no effect of BAS (Table 1).

**Table 1:** The effects of bile acid sequestration (BAS) on morphological, plasma and hepatic parameters in lean and diabetic mice.

	L	L BAS	db	db BAS
Body weight (g)	23.9±1.1	24.9±0.9	37.9±3.2†	39.7±3.5†
Liver weight (g)	1.14±0.08	1.19±0.09	1.74±0.24†	1.93±0.27†
Liver Weight/Body weight (%)	4.7±0.2	4.7±0.2	4.6±0.6	4.9±0.7
**Plasma Parameters**	21.3±0.9	20.9±0.8	21.9±3.1	20.9±3.1
4 hour fasted blood glucose (mmol/L)	8.3±0.6	8.8±0.9	27.7±4.4†	19.3±4.8* †
Blood glucose at 6 hour of MIDA infusion (mmol/L)	9.2±0.5	9.7±1.0	32.7±3.9 #	23.1±3.9* †
4 hour fasted plasma insulin (mU/L)	5.7±1.5	7.2±1.4	44.1±26.3†	49.8±19.3†
Plasma insulin at 6 hour of MIDA infusion (mUl/L)	6.7±1.8	7.5±1.6	66.6±23.3†	48.0±18.4†
HOMA Index	1.0±0.3	1.2±0.3	34.8±10.1†	16.9±6.7* †
NEFA	300±109	393±92	697±149†	555±149
Lacate (mmol/L)	8.4±1.1	8.3±1.3	6.8±1.5	8.6±1.8
3-Hydroxybutyrate (mM)	0.28±0.16	0.28±0.16	1.5±0.95*	0.70±0.27
Triglycerides (mmol/L)	0.74±0.16	0.52±0.11*	0.99±0.14	0.67±0.14†
Total cholesterol (mmol/L)	2.3±0.3	2.3±0.2	2.8±0.1†	3.2±0.4* †
**Liver Parameters**				
Triglycerides (µmol/L)	16±6	31±14*	44±11†	59±21* †
Glycogen (µmol/g liver)	62±45	47±30	175±21†	216±37†
Glucose-6-Phosphate (nmol/mg liver)	0.69±0.18	0.74±0.18	0.40±0.16†	0.42±0.10†
Free fatty acids (nmol/g liver)	366±225	597±315	3079±1503†	4406±2013†

7 h fasted parameters (unless otherwise stated) in lean mice (L), lean mice supplemented with BAS (L BAS), *db/db* (db) and *db/db* mice supplemented with BAS (db BAS). Data are shown as means ± SD;

*p<0.05 vs. same genotype,

† p<0.05 vs. lean same condition.

### Bile acid sequestration increased metabolic clearance of glucose without affecting hepatic glucose production

BAS promotes specific changes in hepatic cholesterol and bile acid synthesis [24]. To gain insight in hepatic glucose metabolism upon BAS-treatment, *in vivo* glucose metabolism was studied in lean and *db/db* mice after 2 weeks of BAS treatment. First, whole body and hepatic glucose metabolism in *db/db* mice was characterized.

The peripheral tissue disposal of plasma glucose (also regarded as the amount of glucose taken up by tissues) was significantly higher in *db/db* mice compared to lean mice (93.1.±13.1 vs. 117.0±14.6 µmol.kg^−1^.min^−1^, lean *vs. db/db*, Table 2), but the metabolic clearance rate (volume of blood cleared from glucose) was significantly lower in *db/db* mice (9.9±1.3 vs. 3.6±0.7 ml.kg^−1^.min^−1^, lean *vs. db/db*, Table 2), because the plasma glucose concentrations (amount of glucose per volume) were almost 3 times higher in *db/db* compared to lean mice (9.2±0.5, 32.7±3.9 mmol/l, lean *vs. db/db*, Table 1).

**Table 2:** Effects of BAS on *in vivo* hepatic glucose metabolism.

	L	LBAS	db	db BAS
**Rate of plasma glucose:**				
Peripheral tissue disposal (µmol/kg/min)	93±13	107±9.2	117±13†	119±8†
Metabolic clearance rate (ml/kg/min)	9.9±1.3	10.7±0.6	3.6±0.7†	5.3±0.3† *
	**Contributions to endogenous glucose production rate (µmol/kg/min)**			
*De novo* glucose-6-phosphate synthesis to glucose	60±7	64±8	66±6	56±8*
Glycogen to glucose	27±6	37±5*	49±8†	61±6† *
**Endogenous glucose production rate**	87±13	101±9	115±14†	117±6†
	**Contributions to hepatic glucose production rate (µmol/kg/min)**			
Endogenous glucose production rate	87±13	101±9	115±14†	117±6†
Glucose cycling rate	31±4	38±3	129±53†	156±53†
**Total hepatic glucose production rate**	118±17	139±17	244±54†	273±58†
	**Flux rates (µmol/kg/min)**			
Glucokinase	47±5	56±7	150±58†	184±54†
Glucose-6-Phosphate *de novo* synthesis	86±6	87±9	74±11†	61±7† *

**I** *n vivo* parameters of hepatic glucose metabolism during the last 3 hours of the infusion experiment in lean mice (L), lean mice supplemented with BAS (L BAS), *db/*db mice (db) and *db/db* mice supplemented with BAS (db BAS) are depicted in the table, the graphic Graphic S1 illustrates the individual glucose fluxes for a better understanding of the table in a schematic manner. The second panel of the table entitled “Contributions to endogenous glucose production rate” shows the contribution of *de novo* glucose-6-phosphate synthesis to glucose (displayed as fluxes e+b in Graphic S1) and of glycogen to glucose (displayed as fluxes c+b Graphic S1) which altogether make up the endogenous glucose production rate. The third panel entitled “Contributions to hepatic glucose production rate” takes into account the glucose cycling rate and thus shows the contributions of the endogenous glucose production rate and the glucose cycling rate (which consists of the cycling of glucose: 1. from glucose to glucose-6-phosphate (depicted as a in the graphic, the glucokinase flux) and back (depicted as b in Graphic S1, the glucose-6-phosphatase flux); 2. Glucose-6-phosphate to glycogen and back, fluxes c+d in Graphic S1; and 3. Gluconeogenesis, flux e in the graphic (glycolysis is also a part of this but cannot be measured in the present set up) to the total hepatic glucose production rate which equals the flux rate through glucose-6-phosphatase. The lower panel shows the flux rates through glucokinase (a in the graphic) and the rate of glucose-6-phosphate *de novo* synthesis (c+e in Graphic S1). Each value represents the mean ± SD;

*p<0.05 vs. same genotype untreated;

† p<0.05 vs. L same condition.

A strongly increased hepatic glucose cycling brought about by a massively increased glucokinase flux was observed in *db/db* compared to lean mice (46.7±4.5 *vs.* 150.2±58.3 µmol.kg^−1^.min^−1^, lean *vs. db/db*, Table 2). In *db/db* mice *de novo* synthesis of glucose-6-phosphate was significantly decreased when compared to lean mice (86.0±6.0 µmol.kg^−1^.min^−1^ *vs.* 73.7±10.9 µmol.kg^−1^.min^−1^, lean *vs. db/db*, p<0.05, Table 2). Endogenous glucose production (excluding glucose cycling) was slightly, but significantly, higher (87.3±13.0 vs. 115.0±14.0 µmol.kg^−1^.min^−1^, lean *vs. db/db*, p<0.05, Table 2) whereas total hepatic glucose output (including glucose cycling) was massively higher in *db/db* compared to lean mice (118.0±16.6 µmol/kg^−1^.min^−1^ vs. 243.5±54. µmol/kg^−1^.min^−1^ lean and *db/db* mice, respectively (Table 2).

BAS treatment had differential effects on whole body glucose metabolism in lean and *db/db* mice. Irrespective of the decreased plasma glucose concentration, BAS treatment had no effect on the flux through glucokinase in livers of *db/db* mice. Since the glucokinase flux remained significantly high, glucose cycling remained significantly higher in treated *db/db* mice when compared to treated lean mice. In treated *db/db* mice the rate of endogenous glucose production did not change and the rate of total hepatic glucose output tended to increase, albeit non-significantly (Table 2). Although BAS was not effective to reduce rates of hepatic glucose consumption or production, we did observe a significant decrease of *de novo* glucose-6-phosphate synthesis in treated *db/db* mice (87.4±9.4 µmol/kg^−1^.min^−1^ *vs*. 61.2±7.4 µmol/kg^−1^.min^−1^, lean vs. *db/db* mice, p<0.05, Table 2). This accounted for the significantly decreased contribution of newly synthesized glucose-6-phosphate towards plasma glucose (Table 2). Since the flux through glucokinase and glucose cycling remained significantly high, these decreases of *de novo* glucose-6-phosphate synthesis and newly synthesized glucose-6-phosphate partitioning towards glucose did not translate into a decreased total glucose output in *db/db* mice.

Importantly, while the peripheral glucose disposal (amount of glucose taken up by tissues) was not different in BAS-treated *db/db* mice compared to untreated *db/db* mice, significantly more blood was cleared of glucose (metabolic clearance rate) in BAS-treated *db/db* mice (∼37%, Table 2) as blood glucose concentrations were significantly reduced in -treated *db/db* mice compared to untreated *db/db* mice (∼30%, Table 1) indicating a higher affinity of peripheral tissues to glucose in BAS treated *db/db* mice. Increased glucose uptake of glucose will decrease the glucose concentration until a new steady state is reached at lower glucose concentrations when there is again a balance between plasma glucose appearance and disappearance.

To assess whether changes in hepatic glucose metabolism were in parallel with changes in gene expression patterns, expression levels of genes involved in hepatic glucose metabolism in untreated lean and *db/db* mice were compared. Compared to lean mice, in *db/db* mice expression levels of glucose transporter 2, glucokinase, glucose-6-phosphate hydrolase as well as glucose-6-phosphatase were increased, while expression levels of genes of β-oxidation like acetyl CoA-carboxylase 2 and pyruvate dehydrogenase, were decreased and no difference was observed in the expression of the gene encoding phospho-*enol-*pyruvate carboxykinase nor in glycogen phopshorylase, glycogen synthase, pyruvate kinase and carnitine palmitoyltransferase 1. (Table 3). Additionally, expression levels of these genes were measured in both models following BAS-treatment. BAS treatment had differential effects on expression of genes involved in hepatic glucose metabolism. In lean mice, expression of glucokinase was increased upon BAS treatment whereas expression levels remained high in *db/db* mice. Quite surprisingly, expression of the glycolytic enzyme pyruvate kinase was strongly increased and phospoenolpyruvate carboxylase was decreased upon treatment in *db/db* mice whereas expression levels remained unaffected in lean mice upon treatment. (Table 3). Acetyl CoA-carboxylase 2 expression levels of treated *db/*db mice increased to that observed in lean control mice. In addition, hepatic gene expression levels of fibroblast growth factor 21 (FGF21), which has lately gained attention for its remarkable *in vivo* actions on glucose metabolism, were measured [36]. Surprisingly, expression levels of FGF21 were 2-fold increased in treated lean and 3-fold increased in treated *db/db* mice compared to untreated counterparts (Table 3).

**Table 3:** mRNA expression levels.

	L	LBAS	db	db BAS
**Gluconeogenesis**				
Phosphoenolpyruvate carboxylase	1.0±0.3	0.8±0.2	1.0±0.2	0.7±0.2*
Glyogen phosphorylase	1.0±0.2	0.9±0.2	1.0±0.1	1.0±0.1
Glucose-6-phosphate hydrolase	1.0±0.2	1.2±0.4	2.9±1.5†	3.7±1.1†
Glucose-6-phosphatase	1.0±0.2	1.2±0.4	1.7±0.3†	1.8±0.3†
Glycogen synthase	1.0±0.3	0.8±0.2	0.9±0.1	1.0±0.1
**Glycolysis**				
Glucose transporter 2	1.0±0.3	1.2±0.2	1.4±0.3†	1.6±0.2†
Glucokinase	1.0±0.3	1.7±0.5*	1.8±0.6†	2.3±0.3†
Pyruvate kinase	1.0±0.3	0.9±0.3	1.1±0.2	2.1±0.4† *
**β-oxidation**				
Acetyl CoA-carboxylase 2	1.0±0.2	1.1±0.4	0.5±0.1†	1.0±0.2*
Carnitine palmitoyltransferase 1	1.0±0.6	1.2±0.3	0.9±0.2	0.8±0.1†
Pyruvate dehydrogenase	1.0±0.5	0.8±0.5	0.5±0.2†	0.8±0.2†
Fibroblast growth factor 21	1.0±0.3	2.1±1.0*	0.6±0.2	2.6±0.8*

Hepatic mRNA expression levels of genes involved in hepatic glucose metabolism in lean (L, n = 5), lean mice supplemented with BAS (LBAS, n = 6), *db/db* mice (db, n = 8) and *db/db* mice supplemented with BAS (db BAS, n = 8). Expression of genes was normalized to 18S-rRNA levels. 18S-rRNA levels were similar in livers of all animals. Each value represents the mean ± SD;

*p<0.05 vs. same genotype untreated;

† p<0.05 vs. L same condition.

### Bile acid sequestration reduced long-chain acylcarnitine content in muscle and plasma in db/db mice

**Figure 1:**
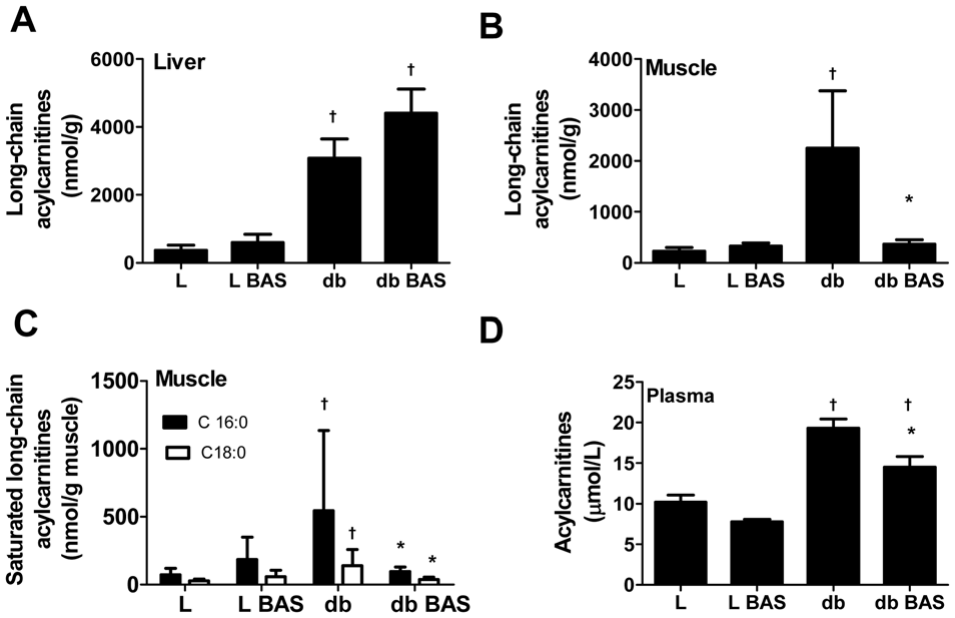
Effect of BAS on liver, skeletal muscle and plasma acylcarnitines. Effect of BAS on hepatic long-chain acylcarnitines content (Sum of C16∶0, C16∶1, C18∶0, C18∶1 and C18∶2) (A), skeletal muscle long-chain acylcarnitines content (B), skeletal muscle saturated long-chain acylcarnitines content (C) and plasma acylcarnitine concentration (D) in lean mice (L, (n = 5), lean mice supplemented with BAS (L BAS, n = 4), db/db mice (db, n = 5) and db/db mice supplemented with BAS (db BAS, n = 5). Data are mean ± SD; † p<0.05 vs. L same condition; * p<0.05 *vs*. same genotype untreated.

Skeletal muscle is the major site of both glucose and fatty acid uptake and oxidation [4]. It is known that under circumstances of high glucose concentrations muscle favors glucose uptake and oxidation over fatty acid uptake and oxidation [4], while accumulating excess fatty acids, in particular saturated free fatty acid species like stearate and palmitate [37], [38]. Recently, it has been shown that excessive fatty acid oxidation in diabetic mice, results in inefficient oxidation [39]. Concomitantly, high intracellular concentrations of long-chain acylcarnitines, markers of inefficient mitochondrial fatty acid oxidation, were measured. High concentrations of these intermediates can impair the switch to carbohydrate oxidation, a marker of “metabolic flexibility”. To study whether BAS affected fatty acid metabolism, long-chain acylcarnitine contents in liver and skeletal muscle were measured. In *db/db* mice, both skeletal and liver long-chain acylcarnitines were strongly increased compared to lean mice (Figure 1). BAS treatment had no effect on hepatic long-chain acylcarnitine content in *db/db* mice (Figure 1A). In contrast, in skeletal muscle of *db/db* mice a strong reduction of long-chain acylcarnitine content was observed which almost reached the level of untreated lean mice (Figure 1B). Specifically saturated long-chain acylcarnitine species of palmitic (C16∶0) and stearic acid (C18∶0) were affected (Figure 1C). Paralelling the BAS effect on skeletal muscle, BAS treatment reduced the high levels of plasma acylcarnitines (Figure 1D) by reducing both short chain and long-chain acylcarntitines *(data not shown).* Apparently, increased metabolic clearance of glucose by peripheral tissue is paralleled by changes in long-chain acylcarnitine content of muscle indicative of increased efficiency of mitochondrial fatty acid oxidation.

## Discussion

The leptin receptor deficient *db/db* mouse is a widely utilized mouse model of type 2 diabetes as it displays several of the features of human type 2 diabetes at 12 weeks of age [40]. However, no characterization describing disturbances of *in vivo* hepatic glucose metabolism in this mouse model is available. Here, we first show that leptin receptor deficiency results in a variety of alterations of *in vivo* hepatic glucose metabolism, the most prominent perturbations being the massively increased glucokinase flux and glucose cycling in *db/db* mice. Secondly, we tested whether the bile acid sequestrant Colesevelam (BAS) induces its reported blood glucose-lowering actions [23], [24] by specific alterations of *in vivo* hepatic glucose metabolism. Our results, however, demonstrate that BAS treatment increased metabolic clearance of glucose by peripheral tissue without affecting hepatic glucose production. Intrigiungly, we observed a decrease in skeletal muscle long-chain acylcarnitine content in treated *db/db* mice. We speculate this lowering in skeletal muscle long-chain acylcarnitines to be indicative of an improved skeletal muscle insulin sensitivity and glucose uptake.

A major and novel observation of this study concerns the disturbances in *in vivo* hepatic glucose metabolism in *db/db* mice. Most strikingly, glucokinase flux was drastically increased in *db/db* mice compared to lean mice. Irrespective the dramatic increase of the HOMA index in *db/db* compared to lean mice, the metabolic clearance of glucose by the liver was essentially not affected. Moreover, a similar ineffectiveness of insulin to modulate hepatic glucose metabolism was apparent from the data on *de novo* synthesis of glucose-6-phosphate: biosynthesis of glucose-6-phosphate was only slightly decreased (∼20%) in *db/db* compared to lean mice. Moreover, fluxes through glucokinase and *de novo* glucose-6-phosphate synthesis were insensitive towards changes in insulin concentration.

Secondly, we studied whole body glucose metabolism. We found the metabolic clearance rate of glucose by peripheral organs was ∼3-fold lower in *db/db* mice at blood glucose concentrations more than 3 times higher and plasma insulin concentrations ∼7.5 times higher than those of lean mice. It is of interest to compare the hepatic clearance of glucose by glucokinase (as derived by dividing blood glucose by the glucokinase flux) with values obtained for peripheral glucose clearance in *db/db* mice. In *db/db* mice, peripheral clearance of glucose was strongly reduced, whereas hepatic clearance of glucose by glucokinase was hardly affected compared to lean mice (5.1±0.7 ml.kg^−1^.min^−1^ vs. 4.6±0.7 ml.kg^−1^.min^−1^, lean vs. *db/db* mice, data not shown in the results section), the observed increase in glucose cycling is most likely driven by the extreme hyperglycemia in *db/db* mice.

Furthermore, the rate of gluconeogenesis was hardly affected by the prevailing high glucose and insulin concentrations in *db/db* mice. Similar observations were made previously by us in *ob/*ob mice [25]. It clearly indicates that the increase in blood glucose concentration in both *db/db* and *ob/ob* mice is driven by an impaired uptake and metabolism of glucose in peripheral organs rather than by increased hepatic glucose production.

Treatment with BAS has been shown to reduce plasma glucose levels in Type 2 diabetic humans [41], [42] and rodents [23], [24]. We tested whether the glucose-lowering actions of BAS in *db/db* mice were due to improvement of disturbed hepatic glucose metabolism. Plasma glucose concentration and *de novo* glucose-6-phosphate synthesis decreased upon BAS treatment in *db/db* mice. Furthermore, insulin concentration tended to decrease, albeit not significantly, when compared to untreated *db/db* mice. The glucokinase flux as well as the glucose cycling rate, however, remained invariantly high. Thus, changes in liver glucose metabolism do not mediate the glucose-lowering effect of BAS.

Intriguingly, we also observed an increase in hepatic triglyceride content in BAS treated *db/db* mice concomitant with an increased hepatic pyruvate kinase expression, likely due to induction of lipogenesis, which we previously described for this model upon BAS treatment [24]. High demand on precursors of cholesterol to bile acids and lipogenesis impairs the TCA cycle. Additionally, phosphoenolpyruvate carboxylase expression was decreased in *db/db* mice upon BAS. Apparently, the flux of carbons and of the TCA cycle needs to be diminished to deliver enough precursors to these biosynthetic processes.

Lipogenesis is often associated with impaired insulin sensitivity. However, to date the relationship between insulin sensitivity and hepatic triglyceride levels is not straight forward and fatty liver does not necessarily result in insulin resistance. For example, fatty liver induced by an Lxr-agonist resulted in improved whole body insulin sensitivity, lowered blood glucose levels and increased metabolic glucose clearance in *ob/ob* mice while increasing hepatic triglyceride levels [43]. Moreover, adding an Lxr-agonist to healthy mice induced hepatic steatosis while not affecting blood glucose levels, whole-body insulin sensitivity nor metabolic clearance rate [43]]. Yet, we found that BAS improved peripheral glucose clearance in *db/db* mice. Interestingly, long-chain acylcarnitine content in *db/db* skeletal muscle, specifically long-chain acylcarnitines of palmitic and stearic acid, decreased upon BAS treatment. Acylcarnitines are by-products of mitochondrial fatty acid oxidation and are formed upon acyl transfer from acyl-CoA to carnitine. Composition and content of acylcarnitines can reflect both high and low rates of mitochondrial fatty acid oxidation. In inborn errors of mitochondrial fatty acid oxidation, in which fatty acid oxidation is impaired, acylcarnitines typically accumulate in tissues and plasma. Additionally, it has been shown that excessive fatty acid oxidation in diabetic mice results in inefficient oxidation. Increased content of long-chain acylcarnitines in skeletal muscle of *db/db* mice compared to lean mice could therefore be indicative of impaired oxidation of long-chain fatty acids in skeletal muscle mitochondria [39]. This results in metabolic inflexibility, *i.e.*, the switch to glucose oxidation cannot be made and insulin is unable to stimulate glucose oxidation [44]. BAS treatment resulted in a clear-cut decrease of long-chain acylcarnitine content of skeletal muscle in *db/db* mice only, nearly to the level that was observed in lean mice. This is suggestive of a more efficient mitochondrial fatty acid oxidation. Concomitantly, a higher metabolic clearance rate of glucose was observed, indicative of an increased ability of the mitochondria to switch to carbohydrate oxidation and indicative of an improved insulin sensitivity. While we report an increased glucose metabolic clearance rate and decreased skeletal muscle content of long-chain acylcarnitines upon BAS treatment in *db/db* mice, we did not observe an effects of BAS treatment on mRNA expression levels of glucose metabolism related genes in peripheral tissues, such as skeletal muscle and white adipose tissue (Table S1).

We also observed an increased ileal expression of glucagon-like-peptide-1 (data not shown), which has been associated with an improved insulin sensitivity [45]. Concomitantly, we also found a BAS-induced massive malabsorption of dietary fatty acids in lean and *db/db* mice (data not shown), indicative of an impaired micelle formation and thus an increased amount o fatty acids passing through the distal parts of the intestine upon BAS treatment. It previously has been described that that fatty acids can induce GLP-1 by stimulation of L-cells in the ileum [46], [47]. Indeed, very recent work by Shang et al showed that 8 week dietary addition of BAS in insulin-resistant, diet-induced obese rats led to an increased Glp-1 release during an oral glucose tolerance test while plasma glucose and insulin concentrations decreased compared to untreated insulin-resistant diet-induced obese rats [48]. The question arises how BAS treatment, which interrupts the enterohepatic circulation of bile acids, can exerts its beneficial effects in peripheral tissues. Bile acids have been shown to increase peripheral energy utilization via activation of the bile acid receptor TGR5 [49]. However, in our previous study we showed that BAS treatment does lower the bile acid concentration in plasma by 30% which should lower rather than increase TGR5 signalling. Intriguingly. We observed that BAS induced a 2-fold increase in hepatic FGF21 gene expression in lean and 3-fold increase in *db/db* mice. Overexpression of FGF21 in livers of *db/db* or *ob/ob* mice or administration of recombinant FGF21 to *db/db* or *ob/ob* mice or to diabetic Zucker rats have been shown to have beneficial effects on insulin sensitivity and glucose clearance due to FGF21 actions on adipose tissue [36], [50], [51]. In addition to FGF21's action on adipose tissue it has recently also been shown to affect muscle tissue [52]. In this respect, FGF21 might provide a link to communicate changes in liver metabolism to peripheral tissues to allow for metabolic adaptation. However, analysis of mRNA expression levels of genes associated with FGF21 in skeletal muscle and white adipose tissue of lean and *db/db* mice treated and not treated with BAS did not provide any further clues herein (Table S2). Thus the role of FGF21 as a link to communicate modulations in liver metabolism and peripheral tissues remains largely elusive at the moment.

In conclusion, this study is the first to characterize hepatic glucose fluxes in *db/db* mice. Glucokinase flux and rates of hepatic glucose output were massively increased in these mice compared to lean mice. Additionally, *db/db* mice had lower metabolic clearance rates of glucose by peripheral tissues compared to lean mice. Decreased plasma glucose levels upon BAS treatment were mainly attributable to increased metabolic clearance of glucose by peripheral tissues: hepatic glucose output remained unaffected. Interestingly, skeletal muscle long-chain acylcarnitine content was decreased in BAS-treated *db/db* mice. Increased hepatic FGF21 gene expression levels might play a role in modulating peripheral glucose handling upon BAS-treatment. This hypothesis, however, requires further investigation.

## Supporting Information

Table S1 **Skeletal muscle and white adipose tissue mRNA expression levels of genes implicated in glucose metabolism.** Skeletal muscle and white adipose mRNA expression levels of genes implicated in glucose metabolism in 7h-fasted lean (L, n = 5), lean mice supplemented with BAS (LBAS, n = 6), *db/db* mice (db, n = 8) and *db/db* mice supplemented with BAS (db BAS, n = 8). Expression of genes was normalized to 18S-mRNA levels. 18S-mRNA levels were similar in tissues.
(DOC)

Table S2 **White adipose and skeletal muscle mRNA expression levels of genes associated with FGF21.** White adipose and skeletal muscle mRNA expression levels of genes associated with FGF21 in 7h-fasted lean (L, n = 5), lean mice supplemented with BAS (LBAS, n = 6), *db/db* mice (db, n = 8) and *db/db* mice supplemented with BAS (db BAS, n = 8). Expression of genes was normalized to 18S-mRNA levels. 18S-mRNA levels were similar in tissues of all animals. Each value represents the mean ± SD; *p<0.05 vs. same genotype untreated; ^†^p<0.05 vs. L same condition.
(DOC)

Graphic S1 **The graphic illustrates the individual glucose fluxes for a better understanding of** **Table 2** **in a schematic manner.** The second panel of the table entitled “Contributions to endogenous glucose production rate” shows the contribution of *de novo* glucose-6-phosphate synthesis to glucose (displayed as fluxes e+b in the graphic) and of glycogen to glucose (displayed as fluxes c+b in the graphic) which altogether make up the endogenous glucose production rate. The third panel entitled “Contributions to hepatic glucose production rate” takes into account the glucose cycling rate and thus shows the contributions of the endogenous glucose production rate and the glucose cycling rate (which consists of the cycling of glucose: 1. from glucose to glucose-6-phosphate (depicted as a in the graphic, the glucokinase flux) and back (depicted as b in the graphic, the glucose-6-phosphatase flux); 2. Glucose-6-phosphate to glycogen and back, fluxes c+d in the graphic; and 3. Gluconeogenesis, flux e in the graphic (glycolysis is also a part of this but cannot be measured in the present set up) to the total hepatic glucose production rate which equals the flux rate through glucose-6-phosphatase. The lower panel shows the flux rates through glucokinase (a in the graphic) and the rate of glucose-6-phosphate *de novo* synthesis (c+e in the graphic).
(DOC)
