# Supplementary material for: Bile Acid Sequestration Reduces Plasma Glucose Levels in db/db Mice by Increasing Its Metabolic Clearance Rate
Source: PLoS One. 2011 Nov 7;6(11):e24564. doi: 10.1371/journal.pone.0024564 (PMC3210115; doi:10.1371/journal.pone.0024564)
Supplement: Table S1 — Skeletal muscle and white adipose tissue mRNA expression levels of genes implicated in glucose metabolism. Skeletal muscle and white adipose mRNA expression levels of genes implicated in glucose metabolism in 7h-fasted lean (L, n = 5), lean mice supplemented with BAS (LBAS, n = 6), db/db mice (db, n = 8) and db/db mice supplemented with BAS (db BAS, n = 8). Expression of genes was normalized to 18S-mRNA levels. 18S-mRNA levels were similar in tissues. (DOC) [file pone.0024564.s001.doc]

**Table S** 1. Skeletal muscle and white adipose tissue mRNA expression levels of genes implicated in glucose metabolism

|  |  |  |  |  |
| --- | --- | --- | --- | --- |
|  | **L** | **LBAS** | **db** | **db BAS** |
| **Skeletal muscle** | | | | |
| Glucose transporter 4 | 1.0 ± 0.2 | 1.1 ± 0.3 | 0.7 ± 0.3 | 0.8 ± 0.3 |
| Carnitine palmitoyltransferase 1B | 1.0 ± 0.1 | 1.0 ± 0.1 | 1.5 ± 0.4† | 1.6 ± 0.5† |
| Carnitine palmitoyltransferase 2 | 1.0 ± 0.2 | 0.8 ± 0.3 | 1.5 ± 0.5 | 1.6 ± 0.5 |
| Acetyl CoA-carboxylase 2 | 1.0 ± 0.1 | 0.9 ± 0.1 | 1.1 ± 0.2 | 1.2 ± 0.2 |
| Acyl-Coenzyme A oxidase 1 | 1.0 ± 0.6 | 0.7 ± 0.1 | 1.1 ± 0.3 | 1.4 ± 0.5 |
| Medium chain acyl co-enzyme-A dehydrogenase | 1.0 ± 0.3 | 0.9 ± 0.1 | 1.7 ± 0.3† | 1.0 ± 0.8† |
| Peroxisome proliferator-activated receptor gamma coactivator-1 alpha | 1.0 ± 0.0 | 1.1 ± 0.2 | 1.3 ± 0.4 | 1.7 ± 0.8 |
| **White adipose tissue** | | | | |
| Acetyl CoA-carboxylase 2 | 1.0 ± 0.3 | 1.5 ± 0.5 | 0.6 ± 0.3 | 1.0 ± 0.5 |
| Acyl-Coenzyme A oxidase 1 | 1.0 ± 0.2 | 1.1 ± 0.5 | 0.7 ± 0.3 | 0.7 ± 0.2 |
| Peroxisome proliferator-activated receptor gamma coactivator-1 alpha | 1.0 ± 0.5 | 1.1 ± 0.4 | 0.4 ± 0.1† | 0.4 ± 0.3 † |

Skeletal muscle and white adipose mRNA expression levels of genes implicated in glucose metabolism in 7h-fasted lean (L, n=5), lean mice supplemented with BAS (LBAS, n=6), *db/db* mice (db, n=8) and *db/db* mice supplemented with BAS (db BAS, n=8). Expression of genes was normalized to 18S-mRNA levels. 18S-mRNA levels were similar in tissues of all animals. Each value represents the mean ± SD; *p<0.05 vs. same genotype untreated; ^†^p<0.05 vs. L same condition
