# Supplementary material for: Bile Acid Sequestration Reduces Plasma Glucose Levels in db/db Mice by Increasing Its Metabolic Clearance Rate
Source: PLoS One. 2011 Nov 7;6(11):e24564. doi: 10.1371/journal.pone.0024564 (PMC3210115; doi:10.1371/journal.pone.0024564)
Supplement: Table S2 — White adipose and skeletal muscle mRNA expression levels of genes associated with FGF21. White adipose and skeletal muscle mRNA expression levels of genes associated with FGF21 in 7h-fasted lean (L, n = 5), lean mice supplemented with BAS (LBAS, n = 6), db/db mice (db, n = 8) and db/db mice supplemented with BAS (db BAS, n = 8). Expression of genes was normalized to 18S-mRNA levels. 18S-mRNA levels were similar in tissues of all animals. Each value represents the mean ± SD; *p<0.05 vs. same genotype untreated; †p<0.05 vs. L same condition. (DOC) [file pone.0024564.s002.doc]

**Table S 2. White adipose and skeletal muscle mRNA expression levels of genes associated with FGF21**

|  |  | |  |  |  |
| --- | --- | --- | --- | --- | --- |
|  | **L** | **LBAS** | | **db** | **db BAS** |
| **White adipose tissue** | | | | | |
| Hormone sensitive lipase | 1.0 ± 0.3 | 1.0 ± 0.3 | | 0.7 ± 0.3 | 0.7 ± 04 |
| Adipose triaglycerol lipase | 1.0 ± 0.3 | 1.0 ± 0.5 | | 0.6 ± 0.2† | 0.7 ± 0.4† |
| Peroxisome proliferator-activated receptor gamma | 1.0 ± 0.2 | 1.4 ± 0.5 | | 0.8 ± 0.2 | 1.0 ± 0.4 |
| **Skeletal muscle** | | | | | |
| Fibroblast growth factor receptor 1 | 1.0 ± 0.2 | 1.0 ± 0.4 | | 1.0 ± 0.5 | 0.8 ± 04 |
| Fibroblast growth factor receptor 4 | 1.0 ± 0.1 | 0.8 ± 0.2 | | 1.5 ± 0.5 | 1.5 ± 0.6 |
| Peroxisome proliferator-activated receptor gamma | 1.0 ± 0.2 | 1.1 ± 0.6 | | 2.2 ± 0.9† | 1.6 ± 0.4† |

White adipose and skeletal muscle mRNA expression levels of genes associated with FGF21 in 7h-fasted lean (L, n=5), lean mice supplemented with BAS (LBAS, n=6), *db/db* mice (db, n=8) and *db/db* mice supplemented with BAS (db BAS, n=8). Expression of genes was normalized to 18S-mRNA levels. 18S-mRNA levels were similar in tissues of all animals. Each value represents the mean ± SD; *p<0.05 vs. same genotype untreated; ^†^p<0.05 vs. L same condition
