# Supplementary material for: Bile Acid Sequestration Reduces Plasma Glucose Levels in db/db Mice by Increasing Its Metabolic Clearance Rate
Source: PLoS One. 2011 Nov 7;6(11):e24564. doi: 10.1371/journal.pone.0024564 (PMC3210115; doi:10.1371/journal.pone.0024564)
Supplement: Graphic S1 — The graphic illustrates the individual glucose fluxes for a better understanding of Table 2 in a schematic manner. The second panel of the table entitled “Contributions to endogenous glucose production rate” shows the contribution of de novo glucose-6-phosphate synthesis to glucose (displayed as fluxes e+b in the graphic) and of glycogen to glucose (displayed as fluxes c+b in the graphic) which altogether make up the endogenous glucose production rate. The third panel entitled “Contributions to hepatic glucose production rate” takes into account the glucose cycling rate and thus shows the contributions of the endogenous glucose production rate and the glucose cycling rate (which consists of the cycling of glucose: 1. from glucose to glucose-6-phosphate (depicted as a in the graphic, the glucokinase flux) and back (depicted as b in the graphic, the glucose-6-phosphatase flux); 2. Glucose-6-phosphate to glycogen and back, fluxes c+d in the graphic; and 3. Gluconeogenesis, flux e in the graphic (glycolysis is also a part of this but cannot be measured in the present set up) to the total hepatic glucose production rate which equals the flux rate through glucose-6-phosphatase. The lower panel shows the flux rates through glucokinase (a in the graphic) and the rate of glucose-6-phosphate de novo synthesis (c+e in the graphic). (DOC) [file pone.0024564.s003.doc]

**Graphic S1**

**
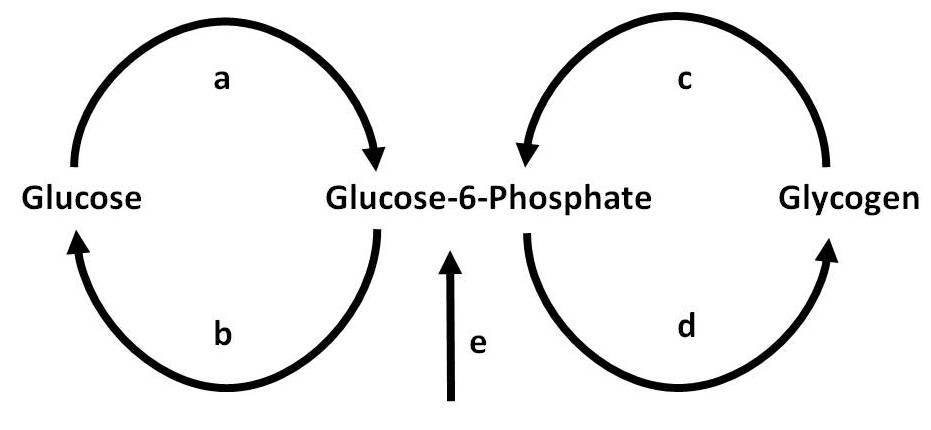
**

The graphic illustrates the individual glucose fluxes for a better understanding of table 2 in a schematic manner. The second panel of the table entitled “Contributions to endogenous glucose production rate” shows the contribution of *de novo* glucose-6-phosphate synthesis to glucose (displayed as fluxes e+b in the graphic) and of glycogen to glucose (displayed as fluxes c+b in the graphic) which altogether make up the endogenous glucose production rate. The third panel entitled “Contributions to hepatic glucose production rate” takes into account the glucose cycling rate and thus shows the contributions of the endogenous glucose production rate and the glucose cycling rate (which consists of the cycling of glucose: 1. from glucose to glucose-6-phosphate (depicted as a in the graphic, the glucokinase flux) and back (depicted as b in the graphic, the glucose-6-phosphatase flux); 2. Glucose-6-phosphate to glycogen and back, fluxes c+d in the graphic; and 3. Gluconeogenesis, flux e in the graphic (glycolysis is also a part of this but cannot be measured in the present set up) to the total hepatic glucose production rate which equals the flux rate through glucose-6-phosphatase. The lower panel shows the flux rates through glucokinase (a in the graphic) and the rate of glucose-6-phosphate *de novo* synthesis (c+e in the graphic).
